# Biologics for pediatric atopic dermatitis: A protocol of a systematic review and meta-analysis

**DOI:** 10.1371/journal.pone.0271712

**Published:** 2023-02-03

**Authors:** Rui Cao, Jiaxing Lu, Zhao Chen

**Affiliations:** 1 North China University of Science and Technology, Hebei, Tangshan, China; 2 Tangshan Gongren Hospital, Hebei, Tangshan, China; University of Catania, ITALY

## Abstract

**Background:**

Atopic dermatitis is a chronic pruritic inflammatory skin disease commonly occurring in children. The objective of this study is to evaluate the treatment of pediatric atopic dermatitis with biologics, as they have displayed immense promising results in several recent clinical trials on atopic dermatitis (AD).

**Methods:**

We will conduct an extensive search for RCTs in several databases, including Embase, Cochrane Library, Web of Science, and PubMed, from the inception of the study till 15th May 2022. The primary outcomes will be the proportion of patients with EASI 75/90/100 after 12–16 weeks of treatment. The secondary outcomes will include the Numerical Rating Scale (NRS), Investigator Global Assessment (IGA)0–1, body surface area (BSA), Dermatology Life Quality Index (DLQI) scores, and incidence of adverse reactions. All studies will be screened by two independent researchers. They will assess the risk of bias in the included studies according to the RCTs bias risk evaluation tool in Cochrane System Review Manual 5.1.0. Meta-analysis will be performed using RevMan V.5.3.0 software.

**Results:**

The research results will provide a reference for the clinical application of biological agents in pediatric atopic dermatitis.

**Conclusion:**

We aim to evaluate the efficacy and safety of biologics in pediatric atopic dermatitis cases and provide evidence-based data for easy clinical application.

**PROSPERO registration number:**

CRD42022319052 (https://www.crd.york.ac.uk/PROSPERO/#joinuppage).

## Introduction

Atopic dermatitis (AD) is a chronic pruritic inflammatory skin disease, with an increasing prevalence globally in all ages; it affects 13% of children and 10% of adults in the United States [1, 2]. This global survey in 18 countries revealed that the prevalence of definite diagnosis of AD in children ranges from 2.7% to 20.1% [2]. Chronic itchy skin caused by AD can have a serious impact on a patient’s quality of life [3, 4]. Although AD can be adequately managed with topical therapies and gentle skin care practices [5], for severe cases, phototherapy, biological agents, and systemic immunosuppressants can be used in conjunction with traditional interventions [6, 7]. In recent years, several studies on the pathogenesis of AD have found that the activation of T helper type 2 (Th2) inflammation-related pathways and its driving factors: interleukin (IL)-4, IL-13, and IL-31, as well as the sequential increase in the inflammatory dendritic epidermal cells, lead to altered cellular responses; thus, predisposing the patient to AD. Such extensive research has led to the development of biologics for treating AD [8, 9]. Dupilumab, as a fully human IL-4Rα monoclonal antibody, can inhibit Th2-mediated inflammatory response by targeting IL-4 and IL-13 that induce inflammatory reactions [10, 11]. Tralokinumab and lebrikizumab are humanized immunoglobulin (IgG4) monoclonal antibodies that inhibit the signaling pathway mediated by IL-13Rα1/IL-4Rα complex through binding to the IL-13α receptor [12]. Omalizumab is a monoclonal antibody used for childhood allergies as it interrupts the IgE-dependent allergic inflammatory cascade by binding to free IgE and preventing its binding to IgE receptors [13]. Etokimab is a human monoclonal IgG1 antibody that neutralizes IL-33 inflammatory responses. Preliminary clinical studies on biologics have demonstrated their efficacy on AD and asthma [14]. Additionally, several I/II clinical trials have revealed afezakinumab, nemolizumab, tezepelumab, secukinumab, GBR 830, and recombinant interferon-γ as emerging treatment options for AD as well other atopic comorbidities in the future [15]. This meta-analysis will systematically evaluate the efficacy and safety of biologics by including a randomized, placebo-controlled study of dupilumab in the treatment of children with AD, and provide an evidence-based reference for its clinical treatment. However, there is a lack of systematic reviews for pediatric AD. Our meta-analysis aims to provide data supporting the clinical application of biologics in treating pediatric atopic dermatitis.

## Methods

This systematic review will follow the PRISMA (Preferred Reporting Items for Systematic Reviews and Meta-Analyses) guidelines. PROSPERO has accepted this protocol for review with the registration number CRD42022319052.

## Ethics and dissemination

The study does not collect raw data and personal information and does not involve jeopardizing the rights of the participants. Ethical approval is not required. The results may be reported in publications and the media.

### Eligibility criteria

#### Study design

All randomized controlled trials (RCTs) that evaluate the efficacy and safety of biologics for treating pediatric atopic dermatitis will be searched thoroughly. Study designs such as non-RCTs and animal studies will be excluded from the review.

#### Types of participants

Patients with atopic dermatitis aged 6 months to 18 years old, regardless of gender, the severity of their condition, and nationality.

#### Types of interventions

Experimental patients will receive a biologic with or without other drugs, while the control patients will receive a placebo or non-biologic treatment.

### Measurement of outcomes

#### Primary outcomes

The primary outcome is the percentage of patients who improved their EASI score from baseline by 75% or 90% or 100% (EASI 75/90/100) based on the decline rate in the Eczema Area and Severity Index (EASI) score.

#### Second outcomes

According to the evaluation standards, the Dermatology Life Quality Index (DLQI), the Numerical Rating Scale (NRS), the Investigator Global Assessment (IGA), body surface area (BSA), and incidence of adverse reactions are evaluated as secondary outcomes.

### Electronic searches

Several databases like Embase, Cochrane Library, Web of Science, and PubMed will be searched from the inception of the study till 15th May 2022 to collect RCTs using biologics for treating pediatric atopic dermatitis. Besides, only English-language articles will be considered.

### Search strategy

The searches will be carried out using a combination of subject headings and keywords. The keywords include “Atopic Dermatitis”, “Atopic Neurodermatitis”, “Upadacitinib”, “Abrocitinib”, “Lebrikizumab”, “Dupilumab”, “Tralokinumab” and “randomized controlled trial”. Table 1 shows the search strategy in PubMed. The same search algorithm will be applied to all other databases.

**Table 1:** Search strategy for pubmed database.

Search strategy in PubMed	
Number	Search items
**1**	Atopic Dermatitides[Mesh]
**2**	Atopic Dermatitides[Title/Abstract]
**3**	Atopic Dermatitis[Title/Abstract]
**4**	Dermatitides, Atopic[Title/Abstract]
**5**	Neurodermatitis, Atopic[Title/Abstract]
**6**	Atopic Neurodermatitides[Title/Abstract]
**7**	Atopic Neurodermatitis[Title/Abstract]
**8**	Neurodermatitides, Atopic[Title/Abstract]
**9**	Neurodermatitis, Disseminated[Title/Abstract]
**10**	Disseminated Neurodermatitides[Title/Abstract]
**11**	Disseminated Neurodermatitis[Title/Abstract]
**12**	Neurodermatitides, Disseminated[Title/Abstract]
**13**	Eczema, Atopic[Title/Abstract]
**14**	Atopic Eczema[Title/Abstract]
**15**	Eczema, Infantile[Title/Abstract]
**16**	Infantile Eczema[Title/Abstract]
**17**	1 OR 2 OR 3 OR 4 OR 5 OR 6 OR 7 OR 8 OR 10 OR 11 OR 12 OR 13 OR 14 OR 15 OR 16 10
**18**	Lebrikizumab[Title/Abstract]
**19**	Omalizumabs[Title/Abstract]
**20**	Tralokinumab[Title/Abstract]
**21**	Etokimab[Title/Abstract]
**22**	Dupilumab[Title/Abstract]
**23**	18 OR 19 OR 20 OR 21 OR 22
**24**	Randomized controlled trial[Title/Abstract]
**25**	Controlled clinical trial [Title/Abstract]
**26**	Randomized [Title/Abstract]
**27**	Randomly [Title/Abstract]
**28**	Clinical Trials [Title/Abstract]
**29**	24 OR 25 OR 26 OR 27 OR 28
**30**	17 AND 23 AND 29

### Data collection and analysis

**Fig 1:**
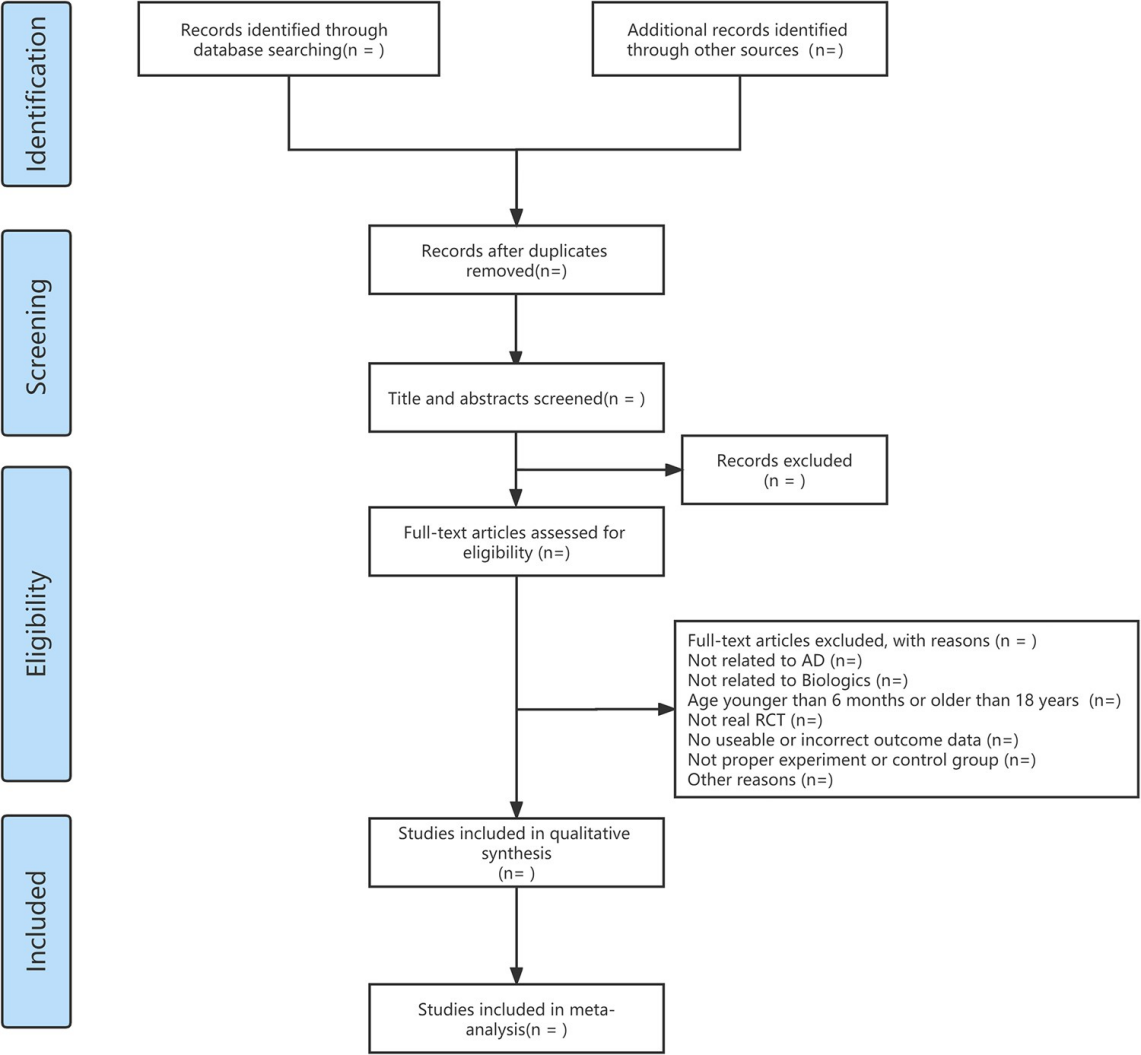
Flow diagram of the study selection process.

Study titles, abstracts, and full texts will be independently reviewed by two researchers (Rui Cao and Jiaxing Lu). We will also record the reasons for excluding irrelevant RCTs. Any disagreements will be settled by a third researcher (Zhao Chen). Fig 1 illustrates the study selection process.

### Risk of bias assessment

Each trial will be evaluated independently by two researchers (Rui Cao and Jiaxing Lu) according to the Cochrane Risk of Bias Tool. We will assess random sequence generation, allocation concealment, blinding of intervention, blinding of outcome assessment, reporting of complete outcome data, selective reporting, and other biases.

There are three levels of study bias: high risk, low risk, and unclear risk.

### Data synthesis and statistical analysis

We will conduct the meta-analysis using RevMan V.5.3.0 software. Continuous data are expressed as standardized mean difference (SMD) with 95% confidence intervals. Dichotomous data will be assessed using the risk ratio (RR) with 95% CIs.

#### Assessment of heterogeneity

To determine statistical heterogeneity, we will calculate I^2^ or use the chi-square test. It will be viewed as low or no heterogeneity if I^2^<50%, while I^2^≥50% will be viewed as significant heterogeneity within the RCTs. If there is no statistical heterogeneity (I^2^ <50% and P> 0.1), the fixed-effects model will be used for meta-analysis. If there is statistical heterogeneity (I^2^> 50% and P < 0.1), the random-effects model will be used for meta-analysis. Consequently, we will conduct a sensitivity or subgroup analysis to identify possible sources of heterogeneity.

#### Subgroup analysis

In cases of insufficient studies, subgroup analysis will be conducted according to different types of biologics, the age range of participants, and the frequency as well as the course of the treatment.

#### Sensitivity analysis

Sensitivity analyses will be performed to check whether the results are robust and reliable, based on methodological quality, sample size, statistical models, and missing data.

#### Assessment of publication bias

The publication bias will be assessed with funnel plots if >10 studies are included in the meta-analysis. The asymmetry of the funnel plot will be checked by Egger’s test.

#### Evidence quality evaluation

The quality of evidence will be categorized as high, moderate, low, or very low, based on their grades.

## Discussion

The expected results may reveal that the efficacy of biological agents in the treatment of pediatric atopic dermatitis is more enhanced than hormonal therapy or immunosuppressants. Additionally, these biological agents display good safety and have little impact on the teenage population.

## Supporting information

S1 Checklist (DOC)

## Abbreviations

AD: atopic dermatitis
BSA: body surface area
CI: confidence interval
DLQI: Dermatology Life Quality Index
EASI: Eczema Area and Severity Index
IGA: Investigator Global Assessment
NRS: Numerical Rating Scale
RCT: randomized controlled trial
RR: risk ratio
SMD: standard mean difference

## References

[1] Barbarot S, Auziere S, Gadkari A, Girolomoni G, Puig L, Simpson EL, et al. Epidemiology of atopic dermatitis in adults: results from an international survey. Allergy. 2018;73(6):1284–93. doi: 10.1111/all.13401 29319189

[2] Silverberg JI, Barbarot S, Gadkari A, Simpson EL, Weidinger S, Mina-Osorio P, et al. Atopic dermatitis in the pediatric population: a cross-sectional, international epidemiologic study. Ann Allerg Asthma Immunol. 2021;126(4):417–28. doi: 10.1016/j.anai.2020.12.020 33421555

[3] Yao T-C, Wang IJ, Sun H-L, Ou L-S, Yu H-H, Wang L, et al. Taiwan Guidelines for the Diagnosis and Management of Pediatric Atopic Dermatitis: Consensus Statement of the Taiwan Academy of Pediatric Allergy, Asthma and Immunology. J Microbiol Immunol Infect. 2022;55(4):561–72. doi: 10.1016/j.jmii.2022.03.004 35487815

[4] Calzavara-Pinton P, Bonamonte D, Marseglia GL, Miraglia Del Giudice M, Musarra A, Nettis E, et al. Diagnosis and management of moderate to severe atopic dermatitis in adolescents. A Consensus by the Italian Society of Dermatology and Venereology (SIDeMaST), the Italian Association of Hospital Dermatologists and Public Health (ADOI), the Italian Association of Hospital and Territorial Allergists and Immunologists (AAIITO), the Italian Society of Allergy, Asthma and Clinical Immunology (SIAAIC), the Italian Society of Pediatric Allergy and Immunology (SIAIP), the Italian Society of Allergological, Occupational and Environmental Dermatology (SIDAPA), and the Italian Society of Pediatric Dermatology (SIDerP). Ital J Dermatol Venereol. 2020;156(2):184–97. 10.23736/S2784-8671.20.06654-7. 32438781

[5] Dennis M, Bhutani T, Koo J, Liao W. Goeckerman therapy for the treatment of eczema: a practical guide and review of efficacy. J DermatolTreat. 2013;24(1):2–6. doi: 10.3109/09546634.2011.607794 21797807 PMC3678985

[6] Boguniewicz M, Fonacier L, Guttman-Yassky E, Ong PY, Silverberg J, Farrar JR. Atopic dermatitis yardstick: practical recommendations for an evolving therapeutic landscape. Ann Allerg Asthma Immunol. 2018;120(1):10–22. doi: 10.1016/j.anai.2017.10.039 29273118

[7] Wollenberg A, Barbarot S, Bieber T, Christen‐Zaech S, Deleuran M, Fink‐Wagner A, et al. Consensus‐based European guidelines for treatment of atopic eczema (atopic dermatitis) in adults and children: part II. J Eur Acad Dermatol Venereol. 2018;32(6):850–78. 10.1111/jdv.14888. 29878606

[8] Boothe D, Tarbox JA, Tarbox MB. Atopic Dermatitis: Pathophysiology. Adv Exp Med Biol. 2017;1027:21–37. doi: 10.1007/978-3-319-64804-0_3 29063428

[9] Boytsov NN, Gorritz M, Wang X, Malatestinic WN, Wade RL, Goldblum OM. The current treatment landscape in adult atopic dermatitis in the United States: results from a cross-sectional real-world study. J DermatolTreat. 2022;33(3):1707–17. doi: 10.1080/09546634.2021.1898530 33752555

[10] Harb H, Chatila TA. Mechanisms of dupilumab. Clin Exp Allerg. 2020;50(1):5–14. doi: 10.1111/cea.13491 31505066 PMC6930967

[11] Rodrigues MA, Nogueira M, Torres T. Dupilumab for atopic dermatitis: evidence to date. Giorn Ital Dermat Venereol. 2019;154(6):696–713. doi: 10.23736/S0392-0488.19.06417-4 31210470

[12] Chiricozzi A, Gori N, Maurelli M, Gisondi P, Caldarola G, De Simone C, et al. Biological agents targeting interleukin-13 for atopic dermatitis. Expert Opin Biol Ther. 2022;22(5):651–9. doi: 10.1080/14712598.2022.2035356 35081849

[13] Yu L, Zhang H, Pan J, Ye L. Pediatric usage of Omalizumab: A promising one. World Allergy Organ J. 2021;14(12):100614. doi: 10.1016/j.waojou.2021.100614 34963793 PMC8672045

[14] Uppal SK, Kearns DG, Chat VS, Han G, Wu JJ. Review and analysis of biologic therapies currently in phase II and phase III clinical trials for atopic dermatitis. J DermatolTreat. 2022;33(2):626–36. doi: 10.1080/09546634.2020.1775775 32507066

[15] Yang N, Chen Z, Zhang X, Shi Y. Novel targeted biological agents for the treatment of atopic dermatitis. BioDrugs. 2021;35(4):401–15. doi: 10.1007/s40259-021-00490-x 34213742

